# Sociodemographic Profile of Blood Donations and Ways to Encourage Them

**DOI:** 10.7759/cureus.60688

**Published:** 2024-05-20

**Authors:** Eduarda Mazurkievz de Freitas, Rômulo Targa Pinto, Arthur Forlin Robert, Kátia Sheylla Malta Purim

**Affiliations:** 1 Medicine, Universidade Positivo, Curitiba, BRA; 2 Medicine, Faculdade Evangélica Mackenzie do Paraná, Curitiba, BRA; 3 Dermatology, Universidade Positivo, Curitiba, BRA

**Keywords:** blood donation, public health, public awareness, socioeconomic factors, blood banks

## Abstract

Introduction: Maintaining blood stocks in Brazil faces challenges; hence, it is crucial to develop effective strategies to recruit and retain donors, such as campaigns and social marketing programs to raise awareness, but there is a lack of studies about the donators' profiles, as well as their barriers or incentives.

Objective: The objective of this study was to analyze the characteristics of donors and the factors that influence their decisions.

Methods: This was a cross-sectional study carried out between April and November 2022 using a structured questionnaire sent through a community created on Facebook, addressing common people over 18 years old, containing closed questions, supported by Google Forms. Statistical analyses were carried out using IBM SPSS Statistics for Windows, Version 17 (Released 2008; IBM Corp., Armonk, New York, United States) and the Chi-square and Fisher's exact tests, with p < 0.05.

Results: The sample relied on 1019 participants, women (72.8%), age group > 30 years (81.1%), the most represented blood type was O positive (37.5%), and men donated more frequently than women (76.5% vs. 40.6%). The main reasons for not donating are medical conditions (39.7%) and lack of time (33.8%). The main reasons for donating are helping voluntarily (97.6%) and donating to family/friends (96.4%).

Conclusion: There was a significant difference between the genders of blood donors, with more men donating, especially those over 30 years old, and with income between 1-8 minimum wages. The main barriers to donating are lack of time and information. Based on this, it is possible to target campaigns at women, young people, and people with income above nine minimum wages. The main reasons for donating are altruism, obtaining test results, and financial benefits.

## Introduction

Maintaining blood donation in Brazil is a constant need. The World Health Organization (2010) recommends that at least 1% of the population be blood donors, while the Ministry of Health points out that the ideal would be to reach 3%; however, the percentage of blood donors was 1.73%, in 2014, and, in 2015, it decreased to 1.58%, reaching the level of 1.4% in 2022 [1]. In 2020 alone, also due to the negative impacts of the coronavirus disease 2019 (COVID-19) pandemic, there was a decrease in the country of approximately 20% in blood donations compared to 2019 [2].

In the current context, it is even more worrying since the blood supply in the vast majority of countries suffers from chronic challenges. In addition to the already known problems related to seasonal blood collection failures and limited blood supplies, these challenges are highly susceptible to intensify as the need for blood products increases due to changing population demographics.

The increase in life expectancy and chronic diseases related to advancing age and the greater complexity of medicine with large-scale medical treatments and interventions, such as transfusions, transplants, oncological procedures, and surgeries, contribute to the growth of blood transfusions and the use of blood products [3]. Simply put, our elderly population will need more blood [4]. Therefore, recruiting new donors and retaining those who have already donated are extremely important to keep blood supplies. Since the only source to collect blood is a living donor, the maintenance of stocks can only be satisfied by a constant flow of volunteer donors.

Unfortunately, the pool of donors is aging along with the general population and this is reflected in a constant increase in the average age of blood donors [5,6]. The National Blood Collection and Use Survey reveals an alarming trend of decreased donations among young donors [5].

Therefore, attracting blood donors is an activity aimed at developing programs that guide the population regarding the importance of voluntary donation [6].

One of the ways to achieve this is the social promotion of awareness and awareness among people about donating blood as an act of citizenship, solidarity, and preservation of human life [7]. Publicizing blood donation in the media has been a strategy used incessantly to reach mainly the population of first-time voluntary donors; however, it is essential to ensure that those who have already donated become recurring donors so that stocks are maintained at minimum quantities of blood and blood components in blood centers [8].

From this perspective, some specific fundraising strategies can be carried out to reach this audience. Social marketing corresponds to the use of a notice/call system through telephone recruitment, mailing, or Internet [9,10]. Another resource used, not only in blood centers but also in basic health units, is reception, a type of institutional strategy considered an effective tool that can provide good service to donors and encourage donor fidelity, is by enhancing the encounter between the user, the professional and the service [11].

These initiatives require the training and goodwill of professionals [3], and studies have shown favorable results when strategies of this nature have been used to attract blood donors [3,8].

In this context, evaluating the effectiveness of the actions undertaken is relevant to determine the choice of policies to be implemented in blood centers and blood banks, as it can identify those capable of enhancing blood donation and corroborate the creation of strategies that aim at continuously improving this process.

Furthermore, projects that present alternative solutions to the lack of blood in hospitals are treated as a health promotion strategy, since it can be considered, as a distinct way of working environment mindset, associated with other policies and technologies developed in the health system that contribute to enhance the public health needs support [12].

Therefore, to collaborate with the improvement of the donor recruitment process, this study aimed to analyze the sociodemographic characteristics and the main barriers associated with blood donation. By understanding the profile of donors and their barriers to donating, opportunities can be identified to improve blood collection programs in a specific way and focus on the main points that negatively affect blood donation and promote public health on a global scale.

## Materials and methods

This is a cross-sectional survey carried out between April and November 2022 using a structured questionnaire containing closed questions supported by the Google Forms platform. The project was approved by the institutional ethics committee (CAAE 45120521.7.0000.0093).

The sample size calculation was 377 (95% CI) participants. The study included people over 18 years of age, of any color/race/ethnicity/gender, who agreed to participate by providing informed consent and completing the questionnaire. People who did not have internet access were excluded, as well as incomplete questionnaires and those who refused to disclose their data. The questionnaire used was based on a previous study [13], including 15 sociodemographic questions, 17 reasons to not blood donations, and 10 reasons/benefits of donating blood. The full questionnaire is available in the appendices. The questionnaire was originally in Portuguese and translated for inclusion in the appendices.

The questionnaire was disseminated through social networks such as Facebook, Instagram, and WhatsApp. In this context, a page called “Blood Donation” was created on the Facebook platform, in which all users were accepted on the page to answer the questionnaire, in addition to receiving instructions and information about blood donation. Subsequently, an analysis was carried out to exclude responses that did not meet the inclusion criteria: accepting to disclose the data and answering the form until the end, totaling 1030 questionnaires answered, with 11 not complying with the prerequisites, thus 1019 responses were computed for the study. 

The minimum sample size was calculated based on the population of the city of Curitiba/Brazil, which is approximately 1,700,000 individuals, with a CI level of 95% and a 5% margin of error, according to Granonik and Hirakata [14] and previous studies [15-17].

The collection of data of interest for the study took place without identifying the participants, in accordance with Resolution No. 466/2012, ensuring respect, secrecy, and confidentiality.

Clinical demographic variables (gender, age group, education, income, blood type, frequency of blood donations) and reasons for donating or not were obtained. The blood group was self-reported according to the classification of the ABO systems (A, B, AB, and O) and Rh Factor (positive or negative). The minimum wage in Brazil in the year 2022 was approximately $250 per month.

Statistical analyses were carried out using IBM SPSS Statistics for Windows, Version 17 (Released 2008; IBM Corp., Armonk, New York, United States) and the Chi-square and Fisher's exact tests, with p < 0.05.

## Results

Of the total of 1030 respondents to the structured questionnaire published on social networks, 11 were excluded based on the criteria listed. The sample showed a predominance of women (72.8%) and an age group over 30 years (81.1%). More than half of the participants had completed higher education (58.5%), with a higher prevalence of income between 1 and 8 minimum wages (67%), considering that 1 minimum wage in Brazil in the year 2022 was approximately $250, the income (1-8 minimum wages) was between $250 and $ 2,000 per month. The most representative blood type was O positive (37.5%), and 47% of those interviewed are frequent donors, having donated more than three times in their lives (Table 1).

**Table 1:** Clinical demographic data of the participants (n=1019)

Demographic data	Variables	Total n=1019 (100%)
Gender	Female	742 (72.8%)
	Male	277 (27.2%)
Age range	Between 18 and 25 years old	133 (13.1%)
	Between 26 and 30 years old	60 (5.9%)
	> 30 years	826 (81.1%)
Education	Elementary School	43 (4.2%)
	High school	380 (37.3%)
	University education	596 (58.5%)
Income	≤ 1 minimum wage	125 (12.3%)
	Between 1 and 8 minimum wages	683 (67.0%)
	≥ 9 minimum wages	114 (11.2%)
	Prefer not to inform	97 (9.5%)
Blood type	A -	56 (5.5%)
	A+	311 (30.5%)
	AB -	10 (1.0%)
	AB +	45 (4.4%)
	B -	18 (1.8%)
	B+	103 (10.1%)
	O -	94 (9.2%)
	O +	382 (37.5%)
Frequency of blood donations	Never donated	366 (35.9%)
	Between 1-2 times	171 (16.8%)
	≥ 3 times	482 (47.3%)

Demographic data regarding gender, age group, education, and income were compared with the frequency of blood donations.

Among survey respondents, approximately 65% have already donated blood, and more than 35% have donated more than five times. Males donated more blood than females (76.45% vs 59.46%). Furthermore, the number of females who have never donated blood is greater than that of males (40.54% vs 23.55%) (Table 2), showing statistical significance (p=0.019).

**Table 2:** Frequency of blood donations by gender

Donation frequency	Female	Male	Total
Never donated	40.54%	23.55%	35.92%
Yes, 1 time	10.41%	7.97%	9.72%
Yes, 2 times	7.16%	6.88%	7.07%
Yes, 3-4 times	11.49%	11.23%	11.38%
Yes, more than 5 times	30.81%	50.36%	35.92%
Grand total	100%	100%	100%

Regarding the age group of participants, 70% of people aged 30 or over have already donated blood at least once, while 64% of people between 18 and 25 have never donated blood, also presenting statistical relevance (p=0.023).

When analyzing income, the most recurring donors were among people earning 1 and 8 minimum wages, representing 68%. The population between 5 and 8 minimum wages drew attention to the number of donations, donating more than five times (45.73%). On the other hand, the largest portion of people who have never donated blood is among those with an income above nine minimum wages (48.2%), with statistical significance (p=0.013).

When analyzing the participants' education and the frequency of blood donations, there was no statistical difference (p=0.863) comparing primary, secondary, and higher education. Even so, the research revealed that the highest percentage of those who have already donated blood have completed higher education (71.43%). Among those who have never donated, the highest percentage is in the class of respondents with incomplete higher education (41.04%).

The main reasons for not donating or donating blood are described in Table 3 and Table 4. Having a physical or medical condition that prevents donation (39.7%) and lack of time (33.8%) were considered important reasons for not donating blood. On the other hand, voluntarily helping people (97.6%) and donating to family or friends (96.4%) were the most relevant reasons for donating blood.

**Table 3:** Reasons for not donating blood (n=1019)

Reasons not to donate blood	Variables	Total
Having a physical or medical condition that prevents donation	Not an important reason	614 (60.3%)
	Yes, it's an important reason	405 (39.7%)
Maintaining risky sexual practices	Not an important reason	803 (78.8%)
	Yes, it's an important reason	216 (21.2%)
Not knowing where to donate	Not an important reason	769 (75.5%)
	Yes, it's an important reason	250 (24.5%)
Getting a tattoo, piercing or acupuncture	Not an important reason	765 (75.1%)
	Yes, it's an important reason	254 (24.9%)
Being suspicious of the sterility of the material	Not an important reason	840 (82.4%)
	Yes, it's an important reason	179 (17.6%)
Religious reasons	Not an important reason	947 (92.9%)
	Yes, it's an important reason	72 (7.1%)
Having little information about the donation	Not an important reason	828 (81.3%)
	Yes, it's an important reason	191 (18.7%)
Lack of time	Not an important reason	675 (66.2%
	Yes, it's an important reason	344 (33.8%)
Fear of the amount of blood lost	Not an important reason	946 (92.8%)
	Yes, it's an important reason	73 (7.2%)
Do not wish to donate blood	Not an important reason	821 (80.6%)
	Yes, it's an important reason	198 (19.4%)
Fear of getting sick	Not an important reason	933 (91.6%)
	Yes, it's an important reason	86 (8.4%)
Fear of pain	Not an important reason	892 (87.5%)
	Yes, it's an important reason	127 (12.5%)
Fear of the hospital environment	Not an important reason	907 (89.0%)
	Yes, it's an important reason	112 (11.0%)

**Table 4:** Reasons for donating blood (n=1019)

Reasons to donate blood	Variables	Total n=1019 (100%)
Voluntarily helping people	Not an important reason	24 (2.4%)
	Yes, it's an important reason	995 (97.6%)
Encouragement from family and/or friends	Not an important reason	137 (13.4%)
	Yes, it's an important reason	882 (86.6%)
Curiosity	Not an important reason	655 (64.3%)
	Yes, it's an important reason	364 (35.7%)
Donating to family or friends	Not an important reason	37 (3.6%)
	Yes, it's an important reason	982 (96.4%)
Receiving test results (HIV, hepatitis, etc.)	Not an important reason	332 (32.6%)
	Yes, it's an important reason	687 (67.4%)
Example of public persons	Not an important reason	643 (63.1%)
	Yes, it's an important reason	376 (36.9%)
Request during military service	Not an important reason	724 (71.1%)
	Yes, it's an important reason	295 (28.9%)
Fee exemption	Not an important reason	504 (49.5%)
	Yes, it's an important reason	515 (50.5%)
Event discounts	Not an important reason	509 (50.0%)
	Yes, it's an important reason	510 (50.0%)

Among the various issues related to the reasons why individuals do not donate blood, some were compared with each other in an attempt to better understand and find significant data on the main reasons for donating or not donating blood in Brazil.

In this analysis of the data, we first found that there is no variation in the response pattern between men and women regarding whether to donate blood, not representing a statistically significant difference (p=0.22).

However, when focusing only on issues that are barriers for the individual to donate blood, we found that 45.38% of respondents who do not donate blood would be able to donate. These individuals do not have any restrictions, whether physical or medical, they do not lack time, they have no piercings, tattoos, or acupuncture, nor have they engaged in any risky sexual practices. Furthermore, this number would increase by another 13% after one year, as individuals who carried out activities with possibly contaminated needles (tattoo, piercing, acupuncture) would again be eligible for blood donation.

Finally, we also found, with statistically significant relevance (p=0.012), that in the universe of individuals who can donate without any of the restrictions mentioned above, 24.73% do not donate due to the lack of time (approximately one in every four respondents).

## Discussion

The frequency of blood donation by gender, age group, and income was evaluated. It was observed that most study participants were female (72.8%) and that men donated more frequently, with 76.5% of them having already made at least one donation. This suggests a gender discrepancy in the willingness to donate blood. Similar results were found by Cavalcante et al., whose research showed that males were more frequent among blood donors in Brazil [18]. In this context, Calvalcante et al. exposed the possible fear among women of developing anemia, besides the greater number of obligations that women accumulate nowadays, justifying a greater lack of time.

Regarding the age group, people aged 30 or over were more likely to donate blood (68.9%), while those between 18 and 25 years old had a lower donation rate (63.9%). Likewise, the work by Silva et al. demonstrated that the age group between 30 and 39 years old also represented the majority of blood donors [19]. This finding may indicate the need to direct efforts to raise awareness and motivate young people and women to become regular donors to ensure a constant blood supply over time. In this sense, for young people, social networks can be an option, as well as carrying out actions in educational contexts and receiving visits from groups of schoolchildren in hemotherapy services [20].

As presented in the present study, the 5th Bulletin of the Hemotherapy Production Information System released by ANVISA in 2018 revealed that “with regard to the profile of the Brazilian donor, it can be observed in the comparison between 2013 and 2016 the maintenance of the predominance of donors over 29 years old and male” [21].

Regarding income, it was found that among people with income between 1 and 8 minimum wages, almost 70% had already donated blood at least once, while the group with income above nine minimum wages had a donation rate of 48%. It is worth noting that we found no previous studies evaluating the relationship between income and motivation for donating blood. However, it is believed that this result may be associated with issues of access to information and awareness about the importance of donating blood, as well as socioeconomic factors that influence the willingness to donate.

In our study, motivations and impediments to donating blood were also observed. The main reasons that led people to donate blood were "voluntarily helping people" (97.6%) and "donating to family or friends" (96.4%), in line with national [10] and international [3] literature, reflecting the importance of altruistic motivations and solidarity among donors. Furthermore, active listening carried out by the professional provides the opportunity for the donor to express what they know, think, and feel, making it a moment of socialization of knowledge about their needs and how to satisfy them [22].

On the other hand, the main impediments to donating were "having some physical or medical condition that prevents donation" (39.7%) and "lack of time" (33.8%). A population-based study conducted in the United States identified that the exclusion factors for blood donation with the highest prevalence among the general population were cancer, anemia, hepatitis B, tattoos, pregnancy, and diabetes [23]. These results highlight the need to raise awareness among the population about the eligibility criteria for blood donation, avoiding embarrassment and burden on the volunteers and the health system.

Also relevant, almost 37% of our interviewees were positively influenced by public figures. Celebrities have the potential to play a significant role in promoting blood donation, helping to raise public awareness, demystifying concerns and taboos, and motivating individuals to become active donors. However, it is important that these interventions are based on evidence and effective strategies, aiming to maximize their impact and long-term sustainability. With a collaborative approach between celebrities, health organizations, and communities, it is possible to analyze the possibility of joint actions to ensure an adequate blood supply and save lives worldwide [24].

In the study by Covos et al., difficulties with travel and stress due to the time waiting for the donation represented barriers to collection [25]. Donor satisfaction could be improved through effective, upfront information about blood collection services, digital technologies, and process flexibility to accommodate donor schedules.

However, even more valuable data, the present study showed that among individuals who can donate without any of the restrictions mentioned above, 24.73% do not donate due to lack of time. In this way, these individuals could be approached with socio-educational measures, including the benefit guaranteed by Law No. 1,075, of March 27, 1950, which guarantees that civil servants, government officials, or military employees who prove the contribution will be exempt from their labor on the day of blood donation.

The same happens for those who work under the Consolidation of Labor Laws (CLT) regime: the CLT provides, in its article 473, that the employee may stop attending work, without prejudice to salary, for one day, in the case of voluntary blood donation duly proven.

Even more surprising, we found that 45.38% of respondents who do not donate blood would be able to donate. These individuals do not have any restrictions. Furthermore, this number would increase by another 13% after one year, as individuals who carried out activities with possibly contaminated needles (tattoo, piercing, acupuncture) would again be eligible for blood donation. These must be achieved in public campaigns.

Thus, by combining this information, we could think of campaigns to encourage donations with characteristics more focused on the public that currently represents the smallest portion of donors.

Regarding the most commonly reported obstacle to becoming a regular blood donor, France et al. found first to be “'laziness” (19.1%), followed by “fear of needles” (10.5%) [26]. Other temporary situations can interfere with donation. During the COVID-19 pandemic, for example, fear and misinformation permeated the blood donation process among young medical students [13]. Góis et al. also indicated that the mass media present themselves as allies in publicizing blood donation. In the same context, ANVISA (2006) showed that the population complained that there should be a greater number of campaigns so that more people donate blood. In the same study, 50.9% of those interviewed believed that the best way to communicate about the donation would be through television, folders, leaflets, pamphlets, and posters [27].

Research by Góis et al. stated that the exposure of the topic in the media is still ineffective, requiring informative, motivational, and awareness-raising education. For this to happen, it is necessary to deal with topics such as the benefits and risks of donation, dispel myths and beliefs, and provide information about the process, the need for blood, the importance of donation, and the use of collected blood [27].

However, it must be remembered that after this hard work of winning over a donor, the experience must be of quality to obtain their loyalty [28]. Thus, reception actions in the waiting room, specifically, are a good way to achieve this goal, which represents the possibility of making them a repeat donor, that is, someone who regularly and spontaneously comes to the blood center to donate blood at least twice a year [12]. Therefore, it is essential adequate training for professionals involved in this process [28]. 

It is necessary to show donors their importance in this process. It is a cultural and educational issue. We need to change people. They usually go to the blood center when a family member or friend asks them to. We want them to return voluntarily after this experience [12].

Cross-sectional studies have some limitations. The design does not allow definitive cause-and-effect relationships to be established and is subject to selection, response, and confounding biases. Furthermore, statistical associations do not necessarily imply causal relationships. However, one of the strengths of this study is its proximity to real-life situations and the fact that it generates hypotheses for future research using other types of design. Nevertheless, this study provides valuable information about the frequency of blood donation and the factors associated with it. Such information can contribute to effective strategies aimed at increasing the number and loyalty of voluntary donors, ensuring an adequate supply of blood and blood components to meet the needs of the population.

## Conclusions

In addition to finding a significant difference between genders, with more men donating blood, income between 1-8 minimum wages, and over 30 years of age, we could identify the barriers that lead individuals who would be able to donate blood but do not do so: lack of time and lack of information. Based on these results, we drew a profile of the individuals who donate blood the least and their main barriers, thus being able to carry out more targeted social campaigns, with a great potential for converting new donors, namely, women and young people, with an income above nine minimum wages. Furthermore, we also know the reasons that lead the population to donate blood, and these should be emphasized: altruism, receiving test results, and exemption from fees or discount policies.

Regarding how to carry out the campaign, more information is needed about the process, donation points, and waiting time in order to facilitate the donation process and accommodate the donors' agenda. Public policies that make donation more accessible and convenient, as well as the example of public figures, and the use of social networks and digital platforms, can play an important role in increasing the number of voluntary donors.

## Appendices

Blood Donation From the Population's Perspective Informed Consent Form

You are being invited as a volunteer to take part in the research project "BLOOD DONATION FROM THE POPULATION'S PERSPECTIVE". This research is being carried out by medical students Eduarda Mazurkievz de Freitas and Rômulo Targa, under the supervision of Professor Kátia Sheylla Malta Purim. The aim of the research is to analyze the perceived advantages and barriers to blood donation from the population's perspective. It should be noted that the data collected will be kept confidential and in the event of publication, no participant's identity will be revealed, and respect and anonymity will be ensured for their responses. Participants will not be subjected to any physical risks. It should also be noted that there will be no direct benefit to the research participant. The results could be useful for planning and developing educational actions to improve health training. The supervisor can be contacted by e-mail: Kátia Sheylla Malta Purim <kspurim@gmail.com>. This form will be in two signed copies, one of which will remain with the researchers and the other with the participant and will be sent by e-mail after the study. Your participation in this study is voluntary and if you no longer wish to take part in the research you can withdraw at any time and ask for the signed informed consent form to be returned to you. If you have any questions, you can also contact the Research Ethics Committee (CEP) of Universidade Positivo - Rua Professor Pedro Viriato Parigot de Souza 5300, campus headquarters - Ecoville, 2nd floor of the Central Library, Curitiba-PR, telephone (41) 3317-3260 e- mail: <cep@up.edu.br> or the National Research Ethics Committee (CONEP) Esplanada dos Ministérios, bloco G - edifício Anexo - Ala "B" - 1o andar - sala 103 B - CEP 70058-900 Brasília - DF, telefone (61) 3315-2150/3315-3821 - e-mail cns@saude.gov.br. You are not responsible for the expenses necessary to carry out the research. Nor will there be any payment or gratitude for your participation in the research. I understand that I am free to stop participating at any time without justifying my decision. If you do not wish to take part, please tick "NO" and if you agree to take part, please click "YES" below. Thank you in advance!

E-mail:

"I voluntarily agree to participate in this study and, in doing so, I understand that my identity will not be revealed and that my confidentiality will be maintained."

·      Yes

·      No

Sociodemographic data:

What is your age? *

·      I'm between 18 and 21 years old 

·      I'm between 22 and 25 years old 

·      I'm between 26 and 30 years old

·      I'm over 30

Gender: 

·      Female

·      Male 

·      I'd rather not say 

·      Other:

Level of education: 

·      Incomplete primary education 

·      Complete primary education 

·      High school incomplete 

·      Completed high school

·      Higher education incomplete 

·      Higher education completed

·      Postgraduate

Weight: 

·      Less than 50 kg 

·      More than 50 kg

 Blood type: 

·      A+

·      A-

·      B+

·      B- 

·      AB+

·      AB- 

·      O+ 

·      O-

Average family income: 

·      Up to 1 minimum wage

·      Between 1 and 4 minimum wages 

·      Between 5 and 8 minimum wages 

·      Between 9 and 12 minimum wages

·      More than 12 minimum wages 

·      I'd rather not say

 Have you donated blood before? If yes, how many times? 

·      I've never donated

·      Yes, 1 time

·      Yes, 2 times

·      Yes, 3- 4 times

·      Yes, more than 5 times

When was the last time you donated blood? 

·      In the current year

·      1 year ago

·      More than 1 year ago 

·      I've never donated

If you're a donor, where was the last time you gave blood? 

·      Ministry of Health hospitals or blood banks 

·      Hospitals or blood banks of military institutions

·      Private hospitals or blood banks

·      I didn't donate

  According to your last experience of donating blood, do you feel motivated to donate again?

·      Yes

·      No

·      I've never donated

If you are a donor, were you motivated to donate blood by requests made on any social network (Twitter, Facebook, Whatsapp, Instagram, etc.)?

·      Yes

·      No

·      I've never donated

Have you experienced any side effects after donating blood? 

·      Yes

·      No

·      I've never donated

Have you ever seen a public campaign calling on people to donate blood?

·      Yes

·      No

    Do you encourage people close to you to donate blood voluntarily? 

·      Yes

·      No

Have you ever received blood before? 

·      Yes

·      No

Obstacles

Check, according to the questions below, if the reason given is a relevant obstacle preventing you from donating blood, you don't donate for this reason.

I have a physical or medical condition that prevents me from donating: 

·       Yes, it's an important reason

·      It's not an important reason

I engaged in risky sexual practices: 

·      Yes, it's an important reason 

·      It's not an important reason      

I don't know where to donate: 

·      Yes, it's an important reason

·      It's not an important reason

I've had a tattoo, piercing, or acupuncture: 

·      Yes, it's an important reason

·      It's not an important reason

I'm suspicious of the sterility of the material: 

·      Yes, it's an important reason

·      It's not an important reason

For religious reasons: 

·      Yes, it's an important reason

·      It's not an important reason

I have little information about the donation: 

·      Yes, it's a major obstacle

·       It's not an important reason    

Lack of time: 

·      Yes, it's an important reason

·      It's not an important reason

I'm afraid of the volume of blood lost: 

·      Yes, it's an important reason

·      It's not an important reason

No one asked: 

·      Yes, it's an important reason

·      It's not an important reason

I don't think about donating blood 

·      Yes, it's an important reason

·      It's not an important reason

I'm afraid of getting sick: 

·      Yes, it's an important reason 

·      It's not an important reason    

For fear of pain: 

·      Yes, it's an important reason

·      It's not an important reason

The donation makes me weak: 

·       Yes, it's an important reason

·      It's not an important reason

I think the blood is commercialized: 

·      Yes, it's an important reason

·      It's not an important reason

I'm afraid of the hospital environment 

·       Yes, it's an important reason

·      It's not an important reason

My parents, friends or relatives told me not to donate blood: 

·      Yes, it's an important reason

·       It's not an important reason

Reasons/Benefits:

Tick according to how important the reason is to YOU

Voluntarily helping people: 

·       Yes, that would motivate me to donate

·      It wouldn't motivate me

Encouragement from family and/or friends: 

·      Yes, that would motivate me to donate

·      It wouldn't motivate me

Curiosity: 

·      Yes, that would motivate me to donate

·      It wouldn't motivate me  

 Donate to family or friends: 

·       Yes, that would motivate me to donate

·      It wouldn't motivate me

Accompany a person who usually donates: 

·       Yes, that would motivate me to donate

·      It wouldn't motivate me

Receive test results on your state of health (HIV, Hepatitis, etc.) 

·       Yes, that would motivate me to donate

·      It wouldn't motivate me

Examples of public figures: 

·      Yes, that would motivate me to donate

·      It wouldn't motivate me

Request during military service: 

·      Yes, that would motivate me to donate

·       It wouldn't motivate me

Aiming for exemption from entrance exam fees: 

·      Yes, that would motivate me to donate

·      It wouldn't motivate me

Discounts on events: 

·      Yes, that would motivate me to donate

·      It wouldn't motivate me
